# Safety and Efficacy of Rituximab and Cyclophosphamide in a Case of Resistant Acquired Hemophilia A in Course of Chronic Lymphocytic Leukemia

**DOI:** 10.7759/cureus.5630

**Published:** 2019-09-12

**Authors:** Francesco Mazziotta, Nadia Cecconi, Lorenzo Iovino, Giulia Cervetti, Mario Petrini

**Affiliations:** 1 Genomec School of Doctorate, University of Siena, Siena, ITA; 2 Clinical and Experimental Medicine, University of Pisa, Pisa, ITA

**Keywords:** acquired hemophilia, factor viii inhibitor, immunosuppressive treatment, steroids, cyclophosphamide, rituximab

## Abstract

Acquired Hemophilia A (AHA) is a rare disease caused by anti-factor VIII autoantibodies. It is usually characterized by clinically significant bleeding at the onset and requires prompt hemostatic and immunosuppressive therapies. Due to its rarity and the lack of randomized trials, its treatment is a great challenge, especially in the relapse/refractory setting. This report presents the case of a patient diagnosed with chronic lymphocytic leukemia who developed a steroid-resistant AHA, successfully managed with aggressive immunosuppressive therapy.

## Introduction

Acquired Hemophilia A (AHA) is a rare and life-threatening autoimmune disease caused by acquired IgG antibodies against factor VIII (FVIII) with an incidence of 1-4/106 per year and median age of incidence > 60 years old [1]. In patients aged between 20 and 40 years, AHA affects preferentially women because of pregnancy disimmunity, while it is quite rare in children [1].

FVIII is a cofactor of factor IXa in the tenase complex, and a deficiency of FVIII reduces the generation of thrombin on platelet surface [2]. As a consequence, FVIII reduction for all causes represents a major cause of bleeding.

According to the EACH2 registry, only in a minority of patients (6.6%) bleeding is absent at presentation [3]. Most of the patients present with skin or soft tissue spontaneous bleeding without hemarthroses typical of congenital forms [4]. Uncontrolled bleeding can lead to severe morbidity and mortality especially after trauma or surgery [4].

Diagnosis should be suspected in patients with negative family and personal history of bleeding combined with isolated and prolonged activated partial thromboplastin time (aPTT) not corrected after the mixing study. The suspect is confirmed by identifying low FVIII activity and by measuring the titer of FVIII inhibitor [4].

Treatment of AHA focuses on bleeding control, antibody eradication, and whenever possible diagnosis and treatment of the underlying disease [4]. Indeed, even if the disorder is idiopathic in 50% of the patients, it can occur in conjunction with post-partum, many autoimmune diseases, malignancies and drugs [4-5]. Here we present the case of a patient affected by chronic lymphocytic leukemia (CLL)-B who developed acquired hemophilia A during hospitalization for sepsis.

## Case presentation

In September 2015, a 39-year-old woman was hospitalized because of fever of unknown origin. 

At the diagnosis, the patient underwent a full clinical history, physical examination, complete blood counts, blood chemistry (including creatinine clearance, liver function, uric acid, lactate dehydrogenase, and coagulation testing), blood cultures, and total body computed tomography (CT).

The patient gave a history positive for CLL-B, treated in 2013 with four cycles of fludarabine, cyclophosphamide, and rituximab (FCR) regimen. Physical examination revealed only one enlarged neck lymph node and hepatosplenomegaly. Apart from a mildly decreased platelet number, complete blood count was normal. Blood chemistry showed renal and liver dysfunction (increased creatinine and low serum albumin, pseudocholinesterase, and antithrombin), elevated C reactive protein, and procalcitonin. Blood cultures tested all negative, but the CT scan was positive for abdominal lymphoadenopathies and hepatosplenomegaly. Bone marrow biopsy was not diagnostic of CLL progression. Fever was successfully managed with empiric broad-spectrum antibiotics and the patient was followed with active surveillance by monitoring blood counts and chemistry. 

After a week of hospitalization, she developed progressive prolongation of aPTT (58.9 sec, normal range: 25.1-36.5 sec), which was not corrected via mixing with an equal volume of normal plasma at 37°C for 2 hours. Further investigation demonstrated FVIII activity of 34.2% (reference range: 50%-150%) and the presence of FVIII inhibitor (1 Bethesda unit/ml). Since the patient didn’t show any sign of bleeding, she underwent therapy with oral administration of Prednisone (PDN) 1,5 mg/kgbw daily to eradicate the inhibitor. 

**Figure 1:**
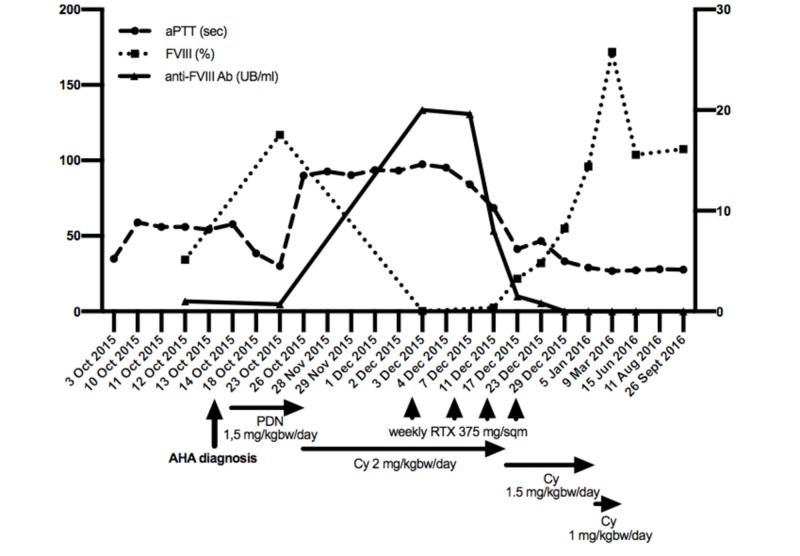
Acquired Hemophilia A (AHA) immunosuppressive treatment and laboratory monitoring Factor VIII and activated partial thromboplastin time (aPTT) trends are plotted on the left y-axis; anti-FVIII Ab is plotted on the right y-axis.

After hospital discharge, she was monitored weekly with complete blood counts and coagulation testing. Despite an initial shortening of aPTT (30.1 sec a week after starting steroid treatment), one month after starting treatment (November 2015) the patient developed diffused skin ecchymosis, metrorrhagia, and again prolonged aPTT (94 sec.). She was hospitalized for a second time, and bleeding was successfully handled with the administration of tranexamic acid and recombinant activated factor VII concentrate (90-120 mcg/kgbw every 2-3 hours till the achievement of effective hemostasis). FVIII activity was 0.2% (reference range: 50-150) and FVIII inhibitor 20 Bethesda unit/ml. Cyclophosphamide (CY) 2 mg/kgbw/day and Rituximab (RTX) 375 mg/sqm once weekly were initiated; a month after (December 2015) aPTT and FVIII levels had normalized and FVIII inhibitor was undetectable. CY dose was reduced to 1.5 mg/kgbw/day and then to 1 mg/kgbw/day in February 2016. In April 2016 treatment was stopped (Fig. 1).

At the last follow-up in November 2018, the patient was still in complete response and she neither reported any new bleeding symptom nor the clinical evidence of CLL relapse. 

## Discussion

CLL is an incurable malignancy of the elderly (less than 2% are younger than 45 years old) [6], and immune dysregulation represents a hallmark of the disease. This substrate, especially in the presence of environmental triggers such as infections, may lead to autoimmunity [7-8]. There are different mechanisms by which pathogens can cause autoimmunity; one of the most studied is molecular mimicry: aminoacid sequence similarities between self-antigen and pathogens may result in the cross-activation of autoreactive T or B cells [8]. Other mechanisms (epitope spreading, bystander activation, cryptic antigens) have also been described [8].

In our case, we couldn’t rule out the persistence of CLL residual disease at the moment of AHA onset. Indeed, our patient showed low platelet count, and immune thrombocytopenia is one of the common autoimmune complications in CLL [9] that often occur at the time of diagnosis or prior to the diagnosis of lymphoproliferative diseases [10]. Moreover, our patient was diagnosed with sepsis, and both low platelet count and lymph node and spleen enlargement could be associated with severe infections rather than to CLL. In our case, these laboratoristic and clinical findings persisted even after sepsis successful management. However, AHA can also occur before the diagnosis or relapse in non-Hodgkin Lymphoma [11].

Limited data [12] are available concerning CLL association with acquired factor VIII inhibitor and, as a consequence, its management. Acquired hemophilia diagnosis and clinical management can be very difficult, especially when there is a need for treatment for the underlying malignancy. Data published [2,13] suggest that treatment or removal of the underlying disorder can aid in removing the inhibitor. In the case of the chronic, incurable underlying disorder as CLL, antibody eradication represents a great challenge for the clinicians. Due to its rarity and the difficulty of performing randomized trials, treatment recommendations are based on data collections such as the EACH2 registry [3,14-16]. AHA treatment is based on controlling acute bleeding with bypassing agents (recombinant activated factor VII or activated prothrombin complex concentrate), replacement therapy (recombinant porcine FVIII, porcine FVIII, and human FVIII when inhibitor titer is below 5 Bethesda unit/ml), or treatment to raise FVIII (desmopressin) [4]. Inhibitor eradication is required usually with immunosuppressive agents (steroids, cyclophosphamide, or rituximab). Other treatments are seldom effective or available [1-2,15]. However, hemostatic and immunosuppressive agents are associated with the risk of infectious and thrombotic side effects [3].

Steroids alone (1 mg/kgbw/day) or in combination with CY (2 mg/kgbw/day) are the most widely used first-line immunosuppressive therapies [15]. This approach is successful in 70%-80% of the cases [17]. The anti-CD20 monoclonal antibody Rituximab is frequently used as a second-line regimen [17] with a success rate of 80%-90% in various anecdotal studies [18-19]. Moreover, EACH2 register showed that the combination of Rituximab with other immunosuppressive agents, such as CY, improves the percentage of complete response [15]. Considering the refractoriness of AHA and the efficacy of Rituximab plus CY in the treatment of autoimmune disorders in CLL [20], we chose the combination of the two agents for our patient.

## Conclusions

In the suspect of AHA, the importance of prompt diagnosis and management cannot be overemphasized. To avoid serious and potentially lethal complications, anti-hemorrhagic and immunosuppressive agents should start as soon as possible after diagnosis. Considering the not-negligible risk of thrombotic and infectious diseases, caution must be exercised on the choice of treatment. However, in young and fit patients, especially in those with relapse of AHA, a more aggressive immunosuppressive approach may be justified. 

Underlying disorders should be sought and eventually treated at diagnosis and during follow-up to achieve long-term responses. 
